# Cs_10_Ta_29.27_O_78_

**DOI:** 10.1107/S1600536809002967

**Published:** 2009-01-28

**Authors:** Martin Zeuner, Alexander Hofer, Wolfgang Schnick

**Affiliations:** a Department Chemie und Biochemie, Ludwig-Maximilians-Universität München, Lehrstuhl für Anorganische Festkörperchemie, Butenandtstrasse 5–13, D-81377 München, Germany

## Abstract

Single crystals of caesium tantalate(V), Cs_10_Ta_29.27_O_78_, were obtained as a serendipitous product in a welded tantalum ampoule by a blank reaction of CsBr and bis­muth subnitrate [Bi_5_O(OH)_9_(NO_3_)_4_] with the container material. The crystal structure of the title compound is made up of a three-dimensional framework constituted by two types of layers, *viz.* (Ta_6_O_15_)_*n*_ and (Ta_3_O_9_)_*n*_, parallel to (001), which are linked together by TaO_6_ octa­hedra (3*m*. symmetry) along [001]. This framework has cavities where three independent Cs^+^ ions (3*m*. and 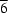 *m*2 symmetry, respectively) are located. The compound reveals a Ta deficiency at one trigonal prismatic coordinated site (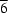 *m*2 symmetry). The composition of the title compound was verified by energy-dispersive X-ray analysis of single crystals.

## Related literature

For a previous powder diffraction study of Cs_10_Ta_29.27_O_78_, see: Michel *et al.* (1978 ▶). For an isotypic compound, see: Haddad & Jouini (1997 ▶). For general background and related structures, see: du Boulay *et al.* (2003 ▶); Magnéli (1953 ▶); Marini *et al.* (1979 ▶); Serafin & Hoppe (1982 ▶).

## Experimental

### 

#### Crystal data

Cs_10_Ta_29.27_O_78_
*M* *_r_* = 7873.51
Hexagonal, 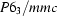

*a* = 7.5170 (11) Å
*c* = 36.340 (7) Å
*V* = 1778.3 (5) Å^3^
*Z* = 1
Mo *K*α radiation
μ = 49.96 mm^−1^
*T* = 294 (2) K
0.03 × 0.02 × 0.01 mm

#### Data collection

Stoe IPDS-I diffractometer
Absorption correction: numerical (*X-SHAPE*; Stoe & Cie, 1999 ▶) *T*_min_ = 0.218, *T*_max_ = 0.607
14043 measured reflections
855 independent reflections
587 reflections with *I* > 2σ(*I*)
*R*_int_ = 0.119

#### Refinement

*R*[*F* ^2^ > 2σ(*F* ^2^)] = 0.033
*wR*(*F* ^2^) = 0.083
*S* = 0.89
855 reflections
67 parameters
Δρ_max_ = 1.54 e Å^−3^
Δρ_min_ = −3.28 e Å^−3^

### 

Data collection: *X-AREA* (Stoe & Cie, 2002 ▶); cell refinement: *X-AREA*; data reduction: *X-AREA*; program(s) used to solve structure: *SHELXS97* (Sheldrick, 2008 ▶); program(s) used to refine structure: *SHELXL97* (Sheldrick, 2008 ▶); molecular graphics: *DIAMOND* (Brandenburg, 1999 ▶); software used to prepare material for publication: *SHELXL97*.

## Supplementary Material

Crystal structure: contains datablocks I, global. DOI: 10.1107/S1600536809002967/wm2217sup1.cif

Structure factors: contains datablocks I. DOI: 10.1107/S1600536809002967/wm2217Isup2.hkl

Additional supplementary materials:  crystallographic information; 3D view; checkCIF report

## Figures and Tables

**Fig. 1.**
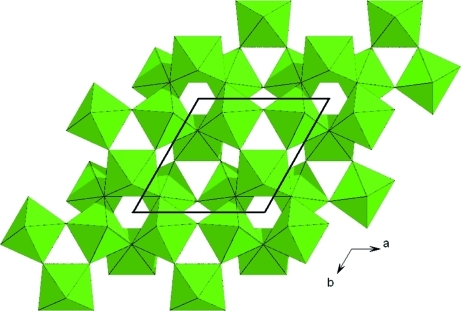
View along [001] on the (Ta6O15)n layer.

**Fig. 2.**
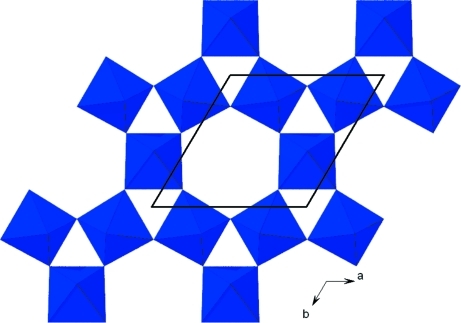
View along [001] on the (Ta3O9)n layer.

**Fig. 3.**
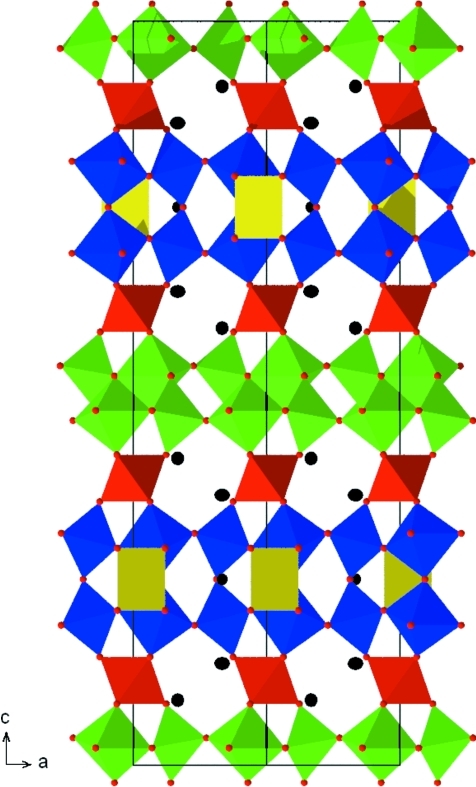
View along [010] on the layers of the title compound connected via TaO6 octahedra. Displacement ellipsoids are drawn at the 50% probability level.

## supplementary crystallographic information

### Comment

The title compound has a three-dimensional framework, constituted by two types of layers, (Ta_6_O_15_)_n_ (Fig. 1, green) and (Ta_3_O_9_)_n_ (Fig. 2, blue), parallel to (001). These layers are linked together along [001], according to the sequence (Ta_6_O_15_)_n_-TaO_6_-(Ta_3_O_9_)_n_-(Ta_3_O_9_)_n_-TaO_6_, by sharing corners of TaO_6_ octahedra (red, Fig. 3). The tantalum atom with deficient occupation is located in a site with trigonal-prismatic coordinated sites (yellow), between two Ta_3_O_9_ units belonging to two neighboring (Ta_3_O_9_)_n_ layers. This framework has cavities which communicate with interconnected channels, parallel to [100]. Cs^+^ ions (black) are located in these cavities. Geometric parameters of the title compound are in the usual ranges. Based on a previous powder diffraction study of Cs_10_Ta_29.27_O_78_ reported by Michel *et al.* (1978), an additional Ta site was found, but was not observed in the current re-investigation. The closely related structure of Tl_10_Nb_29.2_O_78_ was also reported (Marini *et al.*, 1979) with an additional Nb site with too short interatomic distances between the Nb atoms.

The title compound crystallizes isotypically with Rb_5_VONb_14_O_38_ (Haddad & Jouini, 1997), in which the vanadium atom occupies the trigonal-prismatic Ta deficiency site of the title compound. The related compound Cs_3_Ta_5_O_14_ was first investigated by powder diffraction studies by Serafin & Hoppe (1982) and was later re-investigated on the basis of single-crystal X-ray diffraction by du Boulay *et al.* (2003). An overview on the related hexagonal tungsten bronzes of potassium, rubidium and caesium was presented by Magnéli (1953).

### Experimental

The title compound was obtained as a serendipitious product by the reaction of CsBr and bismuth subnitrate (Bi_5_O(OH)_9_(NO_3_)_4_, 98%, Sigma-Aldrich) with the tantalum container material. It was synthesized under argon at temperatures of 1523 K in a welded tantalum ampoule. The air stable title compound crystallizes in brown rods. The chemical composition of the single-crystal was confirmed by energy-dispersive X-ray (EDX) analysis which revealed no impurity elements.

### Refinement

The site occupation factor (s.o.f.) of the Ta-deficient Ta4 site was refined freely. The maximum residual electron density lies 0.95 Å from O1 and the minimum residual electron density lies at the position of the Cs1 atom.

### Crystal data

**Table:**

Cs_10_Ta_29.27_O_78_	*D*_x_ = 7.352 Mg m^−^^3^
*M**_r_* = 7873.51	Mo *K*α radiation, λ = 0.71073 Å
Hexagonal, *P*6_3_/*m**m**c*	Cell parameters from 1275 reflections
Hall symbol: -P 6c 2c	θ = 2.6–31.6°
*a* = 7.5170 (11) Å	µ = 49.96 mm^−^^1^
*c* = 36.340 (7) Å	*T* = 294 K
*V* = 1778.3 (5) Å^3^	Rod, brown
*Z* = 1	0.03 × 0.02 × 0.01 mm
*F*(000) = 3310.4	

### Data collection

**Table:**

Stoe IPDS-I diffractometer	855 independent reflections
Radiation source: fine-focus sealed tube	587 reflections with *I* > 2σ(*I*)
graphite	*R*_int_ = 0.119
ω scans	θ_max_ = 27.5°, θ_min_ = 3.1°
Absorption correction: numerical (*X-SHAPE*; Stoe & Cie, 1999)	*h* = −9→9
*T*_min_ = 0.218, *T*_max_ = 0.607	*k* = −9→9
14043 measured reflections	*l* = −46→46

### Refinement

**Table:**

Refinement on *F*^2^	0 restraints
Least-squares matrix: full	Primary atom site location: structure-invariant direct methods
*R*[*F*^2^ > 2σ(*F*^2^)] = 0.033	Secondary atom site location: difference Fourier map
*wR*(*F*^2^) = 0.083	*w* = 1/[σ^2^(*F*_o_^2^) + (0.0516*P*)^2^] where *P* = (*F*_o_^2^ + 2*F*_c_^2^)/3
*S* = 0.89	(Δ/σ)_max_ < 0.001
855 reflections	Δρ_max_ = 1.54 e Å^−^^3^
67 parameters	Δρ_min_ = −3.28 e Å^−^^3^

### Special details

**Table:**

Geometry. All e.s.d.'s (except the e.s.d. in the dihedral angle between two l.s. planes) are estimated using the full covariance matrix. The cell e.s.d.'s are taken into account individually in the estimation of e.s.d.'s in distances, angles and torsion angles; correlations between e.s.d.'s in cell parameters are only used when they are defined by crystal symmetry. An approximate (isotropic) treatment of cell e.s.d.'s is used for estimating e.s.d.'s involving l.s. planes.
Refinement. Refinement of *F*^2^ against ALL reflections. The weighted *R*-factor *wR* and goodness of fit *S* are based on *F*^2^, conventional *R*-factors *R* are based on *F*, with *F* set to zero for negative *F*^2^. The threshold expression of *F*^2^ > σ(*F*^2^) is used only for calculating *R*-factors(gt) *etc*. and is not relevant to the choice of reflections for refinement. *R*-factors based on *F*^2^ are statistically about twice as large as those based on *F*, and *R*- factors based on ALL data will be even larger.

### Fractional atomic coordinates and isotropic or equivalent isotropic displacement parameters (Å^2^)

**Table:**

	*x*	*y*	*z*	*U*_iso_*/*U*_eq_	Occ. (<1)
Ta1	0.0000	0.0000	0.11749 (3)	0.0166 (2)	
Ta2	0.33551 (10)	0.16775 (5)	0.034126 (15)	0.01525 (19)	
Ta3	0.17146 (6)	0.34292 (11)	0.196383 (17)	0.0204 (2)	
Ta4	0.0000	0.0000	0.2500	0.0163 (8)	0.633 (10)
O1	0.0814 (19)	0.5407 (9)	0.1881 (4)	0.021 (2)	
O2	0.5486 (9)	0.4514 (9)	0.0368 (3)	0.019 (2)	
O3	0.1256 (9)	0.2511 (18)	0.1456 (3)	0.022 (2)	
O4	0.2470 (17)	0.1235 (8)	0.0825 (3)	0.017 (2)	
O5	0.1410 (8)	−0.1410 (8)	0.0233 (3)	0.012 (2)	
O6	0.2379 (18)	0.1190 (9)	0.2082 (3)	0.019 (2)	
O7	0.1878 (15)	0.376 (3)	0.2500	0.023 (4)	
Cs1	0.6667	0.3333	0.2500	0.0337 (6)	
Cs2	0.3333	0.6667	0.08755 (7)	0.0459 (6)	
Cs3	0.6667	0.3333	0.13681 (8)	0.0564 (7)	

### Atomic displacement parameters (Å^2^)

**Table:**

	*U*^11^	*U*^22^	*U*^33^	*U*^12^	*U*^13^	*U*^23^
Ta1	0.0158 (3)	0.0158 (3)	0.0180 (5)	0.00791 (16)	0.000	0.000
Ta2	0.0141 (3)	0.0144 (3)	0.0173 (3)	0.00703 (15)	−0.0001 (2)	−0.00005 (11)
Ta3	0.0181 (3)	0.0217 (4)	0.0226 (4)	0.01083 (19)	−0.00299 (13)	−0.0060 (3)
Ta4	0.0151 (10)	0.0151 (10)	0.0188 (13)	0.0075 (5)	0.000	0.000
O1	0.019 (5)	0.014 (4)	0.031 (6)	0.010 (3)	−0.001 (5)	0.000 (2)
O2	0.020 (4)	0.020 (4)	0.022 (5)	0.013 (5)	0.002 (2)	−0.002 (2)
O3	0.025 (5)	0.019 (6)	0.020 (5)	0.010 (3)	0.000 (2)	0.000 (4)
O4	0.016 (5)	0.016 (4)	0.019 (5)	0.008 (3)	0.007 (4)	0.004 (2)
O5	0.015 (4)	0.015 (4)	0.002 (4)	0.005 (4)	−0.0019 (19)	0.0019 (19)
O6	0.016 (5)	0.013 (4)	0.028 (6)	0.008 (3)	0.000 (4)	0.000 (2)
O7	0.021 (6)	0.038 (11)	0.015 (7)	0.019 (5)	0.000	0.000
Cs1	0.0317 (9)	0.0317 (9)	0.0377 (15)	0.0159 (5)	0.000	0.000
Cs2	0.0443 (8)	0.0443 (8)	0.0493 (15)	0.0221 (4)	0.000	0.000
Cs3	0.0626 (11)	0.0626 (11)	0.0441 (14)	0.0313 (6)	0.000	0.000

### Geometric parameters (Å, °)

**Table:**

Ta1—O3	1.928 (12)	Ta3—O7	1.960 (2)
Ta1—O3^i^	1.928 (12)	Ta3—O6^ii^	2.028 (12)
Ta1—O3^ii^	1.928 (12)	Ta3—O6	2.027 (10)
Ta1—O4^i^	2.050 (11)	Ta3—Ta4	2.9631 (8)
Ta1—O4^ii^	2.050 (11)	Ta4—O6^i^	2.170 (12)
Ta1—O4	2.050 (11)	Ta4—O6^ii^	2.170 (12)
Ta2—O4	1.850 (11)	Ta4—O6^vii^	2.170 (12)
Ta2—O2	1.925 (7)	Ta4—O6	2.170 (12)
Ta2—O2^iii^	1.925 (8)	Ta4—O6^viii^	2.170 (12)
Ta2—O5^ii^	2.070 (4)	Ta4—O6^ix^	2.170 (12)
Ta2—O5	2.070 (4)	Ta4—O7^ii^	2.45 (2)
Ta2—O5^iv^	2.114 (10)	Ta4—O7	2.45 (2)
Ta2—Ta2^v^	3.3049 (10)	Ta4—O7^ix^	2.446 (19)
Ta2—Ta2^iv^	3.3049 (10)	Ta4—Ta3^ii^	2.9631 (8)
Ta3—O3	1.940 (12)	Ta4—Ta3^i^	2.9631 (8)
Ta3—O1	1.941 (12)	Ta4—Ta3^vii^	2.9631 (8)
Ta3—O1^vi^	1.941 (12)		
			
O3—Ta1—O3^i^	94.5 (5)	O3—Ta3—Ta4	113.2 (4)
O3—Ta1—O3^ii^	94.5 (5)	O1—Ta3—Ta4	127.0 (4)
O3^i^—Ta1—O3^ii^	94.5 (5)	O1^vi^—Ta3—Ta4	127.0 (4)
O3—Ta1—O4^i^	173.7 (5)	O7—Ta3—Ta4	55.1 (6)
O3^i^—Ta1—O4^i^	89.8 (3)	O6^ii^—Ta3—Ta4	47.1 (3)
O3^ii^—Ta1—O4^i^	89.8 (3)	O6—Ta3—Ta4	47.1 (3)
O3—Ta1—O4^ii^	89.8 (3)	O6^i^—Ta4—O6^ii^	76.4 (5)
O3^i^—Ta1—O4^ii^	173.7 (5)	O6^i^—Ta4—O6^vii^	138.2 (2)
O3^ii^—Ta1—O4^ii^	89.8 (3)	O6^ii^—Ta4—O6^vii^	138.2 (2)
O4^i^—Ta1—O4^ii^	85.6 (5)	O6^i^—Ta4—O6	76.4 (5)
O3—Ta1—O4	89.8 (3)	O6^ii^—Ta4—O6	76.4 (5)
O3^i^—Ta1—O4	89.8 (3)	O6^vii^—Ta4—O6	88.9 (6)
O3^ii^—Ta1—O4	173.7 (5)	O6^i^—Ta4—O6^viii^	138.2 (2)
O4^i^—Ta1—O4	85.6 (5)	O6^ii^—Ta4—O6^viii^	88.9 (6)
O4^ii^—Ta1—O4	85.6 (5)	O6^vii^—Ta4—O6^viii^	76.4 (5)
O4—Ta2—O2	100.2 (4)	O6—Ta4—O6^viii^	138.2 (2)
O4—Ta2—O2^iii^	100.2 (4)	O6^i^—Ta4—O6^ix^	88.9 (6)
O2—Ta2—O2^iii^	87.5 (7)	O6^ii^—Ta4—O6^ix^	138.2 (2)
O4—Ta2—O5^ii^	89.5 (4)	O6^vii^—Ta4—O6^ix^	76.4 (5)
O2—Ta2—O5^ii^	85.4 (4)	O6—Ta4—O6^ix^	138.2 (2)
O2^iii^—Ta2—O5^ii^	168.9 (4)	O6^viii^—Ta4—O6^ix^	76.4 (5)
O4—Ta2—O5	89.5 (4)	O6^i^—Ta4—O7^ii^	69.09 (12)
O2—Ta2—O5	168.9 (4)	O6^ii^—Ta4—O7^ii^	69.09 (12)
O2^iii^—Ta2—O5	85.4 (4)	O6^vii^—Ta4—O7^ii^	135.5 (3)
O5^ii^—Ta2—O5	100.3 (6)	O6—Ta4—O7^ii^	135.5 (3)
O4—Ta2—O5^iv^	152.4 (5)	O6^viii^—Ta4—O7^ii^	69.09 (12)
O2—Ta2—O5^iv^	99.7 (4)	O6^ix^—Ta4—O7^ii^	69.09 (12)
O2^iii^—Ta2—O5^iv^	99.7 (4)	O6^i^—Ta4—O7	135.5 (3)
O5^ii^—Ta2—O5^iv^	73.2 (4)	O6^ii^—Ta4—O7	69.09 (12)
O5—Ta2—O5^iv^	73.2 (4)	O6^vii^—Ta4—O7	69.09 (12)
O4—Ta2—Ta2^v^	127.6 (2)	O6—Ta4—O7	69.09 (12)
O2—Ta2—Ta2^v^	132.2 (3)	O6^viii^—Ta4—O7	69.09 (12)
O2^iii^—Ta2—Ta2^v^	83.1 (3)	O6^ix^—Ta4—O7	135.5 (3)
O5^ii^—Ta2—Ta2^v^	95.4 (3)	O7^ii^—Ta4—O7	120.000 (1)
O5—Ta2—Ta2^v^	38.3 (3)	O6^i^—Ta4—O7^ix^	69.09 (12)
O5^iv^—Ta2—Ta2^v^	37.37 (10)	O6^ii^—Ta4—O7^ix^	135.5 (3)
O4—Ta2—Ta2^iv^	127.6 (2)	O6^vii^—Ta4—O7^ix^	69.09 (12)
O2—Ta2—Ta2^iv^	83.1 (3)	O6—Ta4—O7^ix^	69.09 (12)
O2^iii^—Ta2—Ta2^iv^	132.2 (3)	O6^viii^—Ta4—O7^ix^	135.5 (3)
O5^ii^—Ta2—Ta2^iv^	38.3 (3)	O6^ix^—Ta4—O7^ix^	69.09 (12)
O5—Ta2—Ta2^iv^	95.4 (3)	O7^ii^—Ta4—O7^ix^	120.000 (1)
O5^iv^—Ta2—Ta2^iv^	37.37 (10)	O7—Ta4—O7^ix^	120.000 (2)
Ta2^v^—Ta2—Ta2^iv^	69.83 (3)	Ta3^ii^—Ta4—Ta3^vii^	135.741 (10)
O3—Ta3—O1	93.3 (4)	Ta3^i^—Ta4—Ta3^vii^	135.741 (10)
O3—Ta3—O1^vi^	93.3 (5)	Ta3—O1—Ta3^x^	140.3 (7)
O1—Ta3—O1^vi^	94.1 (7)	Ta2—O2—Ta2^xi^	151.8 (7)
O3—Ta3—O7	168.3 (7)	Ta1—O3—Ta3	139.9 (7)
O1—Ta3—O7	94.7 (5)	Ta2—O4—Ta1	146.5 (7)
O1^vi^—Ta3—O7	94.7 (5)	Ta2^i^—O5—Ta2	132.0 (6)
O3—Ta3—O6^ii^	88.8 (4)	Ta2^i^—O5—Ta2^v^	104.3 (3)
O1—Ta3—O6^ii^	91.5 (5)	Ta2—O5—Ta2^v^	104.3 (3)
O1^vi^—Ta3—O6^ii^	173.9 (5)	Ta3^i^—O6—Ta3	145.0 (6)
O7—Ta3—O6^ii^	82.4 (6)	Ta3^i^—O6—Ta4	89.8 (3)
O3—Ta3—O6	88.8 (4)	Ta3—O6—Ta4	89.8 (3)
O1—Ta3—O6	173.9 (5)	Ta3—O7—Ta3^vii^	167.5 (11)
O1^vi^—Ta3—O6	91.5 (5)	Ta3—O7—Ta4	83.8 (6)
O7—Ta3—O6	82.4 (5)	Ta3^vii^—O7—Ta4	83.8 (6)
O6^ii^—Ta3—O6	82.9 (6)		

Symmetry codes: (i) −*x*+*y*, −*x*, *z*; (ii) −*y*, *x*−*y*, *z*; (iii) −*y*+1, *x*−*y*, *z*; (iv) *x*−*y*, *x*, −*z*; (v) *y*, −*x*+*y*, −*z*; (vi) −*y*+1, *x*−*y*+1, *z*; (vii) *x*, *y*, −*z*+1/2; (viii) −*y*, *x*−*y*, −*z*+1/2; (ix) −*x*+*y*, −*x*, −*z*+1/2; (x) −*x*+*y*, −*x*+1, *z*; (xi) −*x*+*y*+1, −*x*+1, *z*.
